# Real-world pathogen spectrum, clinical actionability, and host correlates of first-time mNGS testing in hospitalized patients with hematologic diseases

**DOI:** 10.3389/fcimb.2026.1900843

**Published:** 2026-08-27

**Authors:** Ziyi Li, ZiHui Dong, LeMin Jiao, YuDie Cai, YangMiao Zhang, HaiTing Wei, TianQi Fang, ZiYang Zhang, Hang Liu

**Affiliations:** 1 Gene Hospital of Henan Province, Precision Medicine Center, The First Affiliated Hospital of Zhengzhou University, Zhengzhou University, Zhengzhou, China; 2 State Key Laboratory of Metabolic Dysregulation & Prevention and Treatment of Esophageal Cancer, Tianjian Laboratory of Advanced Biomedical Sciences, School of Convergence Medicine, Zhengzhou University, Zhengzhou, China

**Keywords:** clinical actionability, fungal infection, hematologic disease, infection, metagenomic next-generation sequencing, viral detection

## Abstract

**Background:**

Patients with hematologic diseases are highly susceptible to infection. Conventional tests often have low sensitivity. Metagenomic next-generation sequencing (mNGS) can detect many pathogens at once, but its clinical value depends on how the results are interpreted. It is often hard to tell true infection from colonization or contamination.

**Methods:**

We retrospectively studied hospitalized hematologic patients Only adult patients (≥18 years) who received mNGS for the first time. Detected organisms were reclassified using a clinical actionability system. We also analyzed the relationships between mNGS findings, host characteristics, and short-term outcomes.

**Results:**

A total of 134 patients were included. At least one organism was detected in 87.3% of patients, but only 58.2% had highly actionable results. Bacteria were the most common findings, followed by viruses and fungi. Mixed detections were frequent. Actionable results were seen more often in respiratory specimens than in blood specimens. Viral detection was associated with immune status. Pathogen read counts were only weakly related to inflammatory markers and did not independently predict adverse outcomes. Age was the only independent risk factor for adverse outcome.

**Conclusion:**

mNGS had a high detection rate in hematologic patients, but not all positive findings were clinically important. Result interpretation should take specimen type and host status into account. Pathogen read counts alone were not useful for predicting short-term outcome.

## 1 Introduction

Infection is a frequent and serious problem in patients with hematologic diseases. Their susceptibility is related to neutropenia, cytotoxic chemotherapy, corticosteroid use, transplantation, mucosal barrier damage, and impaired immune function (Chiu and Miller, 2019; Gu et al., 2019). In many cases, the clinical signs are not specific. Standard microbiological tests may also fail to identify the cause in time, particularly after prior antimicrobial treatment (Chiu and Miller, 2019; Gu et al., 2019; Han et al., 2019).

Metagenomic next-generation sequencing (mNGS) has emerged as a useful tool for pathogen detection. Unlike conventional methods, it does not rely on culture and can identify bacteria, viruses, fungi, and parasites in a single test (Chiu and Miller, 2019; Gu et al., 2019). This is particularly relevant in immunocompromised patients, who often present with opportunistic infections, mixed infections, or viral reactivation. Several studies have shown that mNGS may increase the diagnostic yield in patients with hematologic diseases and other immunocompromised populations (Zinter et al., 2019; Shen et al., 2021; Wang et al., 2022; Zhang et al., 2022; Yuan et al., 2024). However, a positive result does not always indicate clinically meaningful infection.

A central challenge is how to interpret the result. mNGS can identify true pathogens, but it can also detect colonizing organisms, contaminants, latent viruses, or low-level background sequences (Chiu and Miller, 2019; Gu et al., 2019; Han et al., 2019; Hogan et al., 2021; Vinh Dong et al., 2024). The clinical meaning of the same organism may differ by specimen type and host condition. This is a common problem in hematologic patients. For example, invasive fungal infection and herpesvirus reactivation are not rare in this population, but not every positive result supports a change in treatment (Alexander et al., 2021; Dadwal et al., 2021).

In this study, we reviewed first-time mNGS testing in hospitalized patients with hematologic diseases. We described the spectrum of detected organisms and reclassified each finding according to its clinical actionability. We also examined the associations of actionable findings, fungal and viral detection, and host characteristics with short-term clinical outcomes.

## 2 Methods

### 2.1 Study design and study population

This retrospective single-center study included hospitalized patients with hematologic diseases who underwent mNGS testing for suspected infection. Only the first mNGS result for each patient was analyzed.

### 2.2 Clinical data

Clinical variables were retrieved from the cleaned baseline dataset. These included age, sex, disease stage, blood counts, inflammatory markers, lymphocyte subsets, specimen type, length of hospital stay, and in-hospital outcome. Missing laboratory data had been handled before the present analysis according to clinically defined imputation rules.

### 2.3 mNGS data processing

All mNGS testing was performed in-house at the Precision Medicine Center, The First Affiliated Hospital of Zhengzhou University, as part of routine clinical care. This retrospective study analyzed routinely collected clinical data, and all study procedures were approved by the Institutional Ethics Committee. The original clinical mNGS reports were retrieved from the electronic medical records and imported from the source database for analysis. Microorganism names and corresponding read counts reported by the mNGS assay were extracted from each report. In this study, read count was defined as the number of high-quality sequencing reads assigned to a specific organism after quality control and host sequence removal during the clinical mNGS workflow.

The clinical mNGS reporting process included specimen processing, nucleic acid extraction, library preparation, high-throughput sequencing, bioinformatic analysis, and microbial identification based on alignment against a curated pathogen reference database. Organisms reported by the clinical laboratory were determined according to sequencing evidence, background contamination assessment, and clinical relevance. When multiple organisms were identified within a single specimen, each organism was recorded as an independent finding in the analysis dataset. Organism names were standardized according to current taxonomic nomenclature and classified into five groups: bacteria, viruses, fungi, parasites, and others. Specimen types were categorized as blood, respiratory specimens, cerebrospinal fluid, sterile fluid or tissue, and other specimen types.

### 2.4 Assessment of clinical actionability

Because not all organisms detected by mNGS are clinically relevant, each finding was retrospectively reassessed using a predefined rule-based framework developed for this study. The assessment integrated the known pathogenicity of the detected organism, the specimen source, and the concordance between the mNGS result and the patient’s clinical presentation. Classification was performed by the study investigators using the microbiological and clinical information available in the electronic medical records.

Each detected organism was assigned to one of three actionability categories: high-confidence actionable, intermediate-confidence actionable, or low-confidence/non-actionable. High-confidence actionable findings were considered highly clinically relevant and consistent with the patient’s clinical syndrome. Intermediate-confidence actionable findings had partial microbiological or clinical support but required further clinical correlation. Low-confidence or non-actionable findings were considered more likely to represent contamination, colonization, or microorganisms of limited clinical significance. Representative examples for each category are provided in **Table 1**. For patient-level analyses, each patient was classified according to the highest actionability category among all detected organisms.

**Table 1:** This table summarizes the predefined criteria used to classify organism-level mNGS detections into three actionability tiers based on organism pathogenicity, specimen context, and clinical concordance.

Tier	Definition	Representative examples
Tier 1	Sterile site + typical pathogen + clinical match	CMV viremia in HSCT recipient; Pneumocystis jirovecii with compatible imaging
Tier 2	Partial match	Low-abundance Candida spp.; Human herpesvirus 6 (HHV-6) without compatible syndrome
Tier 3	Likely contaminant	Skin flora in blood; oral commensals in respiratory specimen

Representative examples are provided to enhance reproducibility and support standardized interpretation.

### 2.5 Statistical analysis

Statistical analyses were performed using R version 4.4.2. Continuous variables are reported as median and interquartile range (IQR), and categorical variables as counts and percentages. Group differences were assessed using the Wilcoxon rank-sum test or Kruskal–Wallis test for continuous variables and the chi-square test or Fisher’s exact test for categorical variables, as appropriate.

Spearman correlation analysis was used to evaluate the relationships between mNGS read counts and clinical parameters. P values were adjusted for multiple comparisons using the Benjamini–Hochberg false discovery rate method.

Variables considered clinically important or showing possible associations in exploratory analyses were further examined in linear regression models to identify factors independently associated with mNGS read counts. For exploratory analyses, the maximum pathogen read count detected in each sample was log-transformed before correlation and regression analyses to reduce skewness. Regression coefficients (β) with 95% confidence intervals (CIs) were reported. Logistic regression was used for binary outcomes. Because in-hospital death was infrequent, prognostic analyses were based on a composite adverse outcome of discharge against medical advice.

Multivariable logistic regression was also used to identify factors associated with actionable mNGS findings. Variables were selected according to clinical relevance. Odds ratios (ORs) with 95% confidence intervals (CIs) were reported. Continuous variables were entered as linear terms. IL-6 was log-transformed to reduce skewness. All tests were two-sided, and P < 0.05 was considered statistically significant.

## 3 Results

### 3.1 Cohort characteristics and specimen distribution

**Figure 1:**
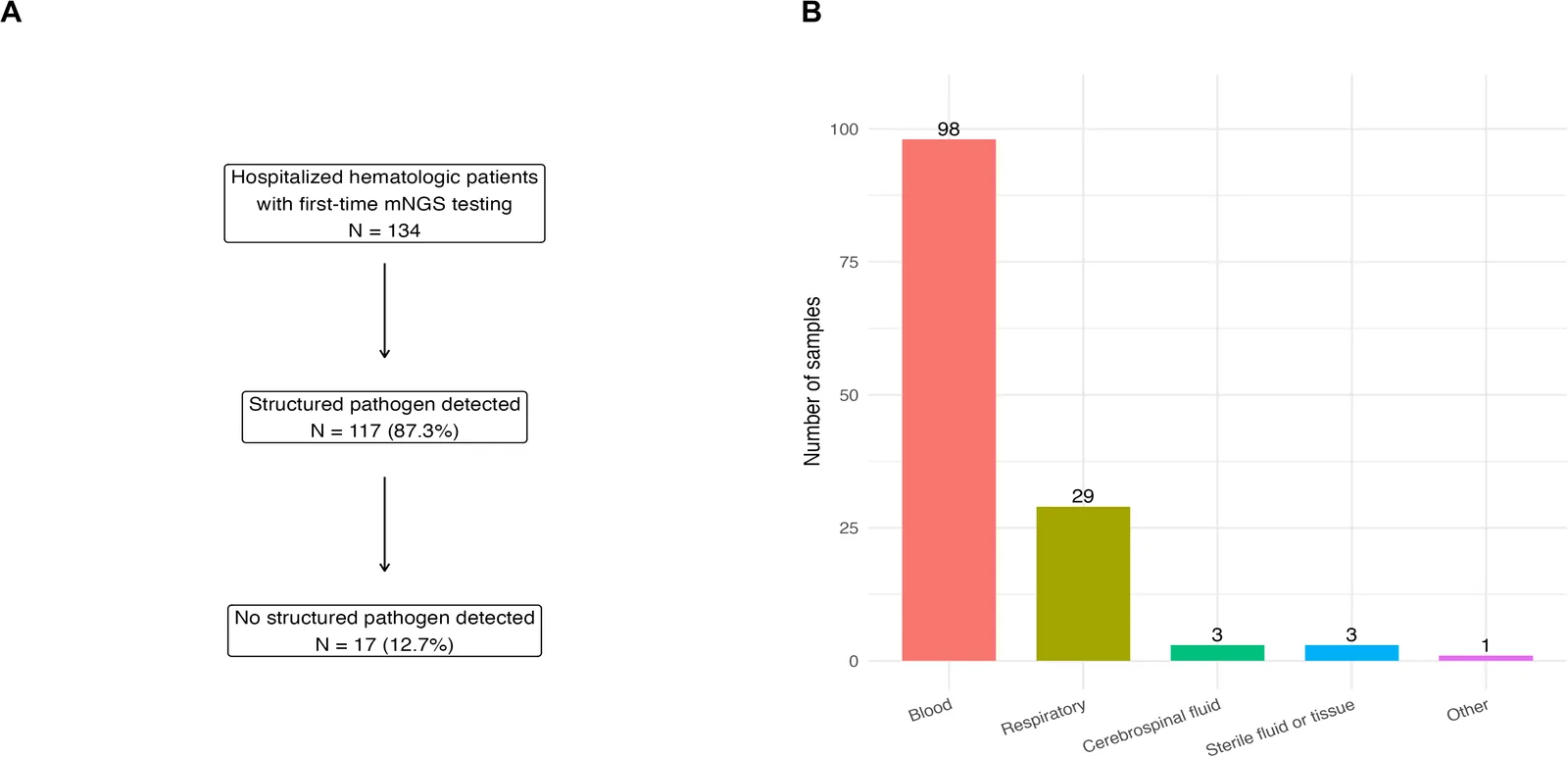
Study flow and specimen distribution. Panel **(A)** shows cohort assembly and structured parsing of first-time mNGS reports. Panel **(B)** shows the distribution of specimen groups in the study cohort.

A total of 134 hospitalized patients were included. After structured parsing of the mNGS reports, 117 patients (87.3%) had at least one detectable organism, while 17 (12.7%) had no structured pathogen identified. Blood was the most common specimen type, accounting for 98 of 134 samples (73.1%). Respiratory specimens were the second most common (29/134, 21.6%). Cerebrospinal fluid and sterile fluid or tissue samples were rare. Baseline characteristics stratified by mNGS positivity are shown in **Table 2**. Cohort assembly and specimen distribution are shown in **Figure 1**.

**Table 2:** Baseline characteristics by structured mNGS positivity.

Variable	level	Overall	Negative	Positive	P-value
Age	NA	42.00 [33.00, 55.75]	49.00 [42.00, 55.00]	41.00 [33.00, 56.00]	0.36
Length of stay (days)	NA	27.00 [17.25, 35.75]	32.00 [27.00, 49.00]	26.00 [17.00, 34.00]	0.01
WBC count (×10^9^/L)	NA	1.15 [0.30, 5.58]	0.72 [0.22, 4.00]	1.50 [0.34, 5.60]	0.38
Neutrophil count, 10^9/L	NA	0.61 [0.07, 2.95]	0.24 [0.07, 0.75]	0.68 [0.08, 3.40]	0.23
Lymphocyte count, 10^9/L	NA	0.35 [0.12, 0.85]	0.23 [0.07, 1.01]	0.37 [0.13, 0.81]	0.59
PCT, ng/mL	NA	0.23 [0.14, 0.60]	0.27 [0.16, 0.78]	0.22 [0.13, 0.60]	0.26
CRP, mg/L	NA	69.04 [40.50, 123.52]	55.52 [30.83, 78.78]	71.49 [42.00, 125.00]	0.52
ESR	NA	44.00 [36.00, 56.00]	48.00 [36.00, 56.00]	44.00 [36.00, 56.00]	0.60
IL-6, pg/mL	NA	89.20 [37.00, 145.00]	83.00 [37.00, 163.00]	91.00 [37.00, 145.00]	0.52
Serum Ferritin	NA	1170.00 [980.00, 1417.50]	1260.00 [980.00, 1440.00]	1170.00 [980.00, 1350.00]	0.66
T lymphocytes, %	NA	72.20 [68.43, 77.10]	71.60 [68.40, 77.85]	72.80 [68.88, 76.80]	0.95
Helper T cells, %	NA	30.00 [15.70, 36.30]	31.40 [27.81, 44.00]	30.00 [15.70, 34.70]	0.19
Suppressor T cells, %	NA	42.00 [35.55, 59.25]	37.73 [36.00, 58.60]	42.00 [35.40, 59.30]	0.52
NK cells, %	NA	9.00 [5.71, 11.00]	7.00 [6.00, 11.00]	9.00 [5.50, 11.00]	0.98
B cells, %	NA	5.60 [1.50, 7.40]	6.00 [2.30, 7.00]	5.60 [1.50, 7.80]	0.93
CD4/CD8 ratio	NA	0.71 [0.26, 1.11]	0.76 [0.45, 1.22]	0.71 [0.26, 1.03]	0.19
CEA	NA	1.88 [1.70, 2.12]	2.12 [1.70, 2.24]	1.88 [1.70, 2.12]	0.39
Sex	Female	54 (40.3%)	10 (58.8%)	44 (37.6%)	0.12
	Male	80 (59.7%)	7 (41.2%)	73 (62.4%)	0.12
Disease stage	Bone-Marrow-Suppression	28 (20.9%)	4 (23.5%)	24 (20.5%)	0.92
	Consolidation	3 (2.2%)	0 (0.0%)	3 (2.6%)	0.92
	Induction-Remission	33 (24.6%)	5 (29.4%)	28 (23.9%)	0.92
	Initial-Diagnosis	11 (8.2%)	0 (0.0%)	11 (9.4%)	0.92
	Non-Remission	3 (2.2%)	1 (5.9%)	2 (1.7%)	0.92
	Post-Transplant	27 (20.1%)	4 (23.5%)	23 (19.7%)	0.92
	Refractory	8 (6.0%)	1 (5.9%)	7 (6.0%)	0.92
	Relapse	8 (6.0%)	1 (5.9%)	7 (6.0%)	0.92
	Remission	3 (2.2%)	0 (0.0%)	3 (2.6%)	0.92
	Unknown	10 (7.5%)	1 (5.9%)	9 (7.7%)	0.92
Initial diagnosis	No	90 (67.2%)	12 (70.6%)	78 (66.7%)	1.00
	Yes	44 (32.8%)	5 (29.4%)	39 (33.3%)	1.00
Newly diagnosed	automatic-discharge	38 (28.4%)	4 (23.5%)	34 (29.1%)	0.83
In-hospital outcome	Death	2 (1.5%)	0 (0.0%)	2 (1.7%)	0.83
	Improved-discharge	94 (70.1%)	13 (76.5%)	81 (69.2%)	0.83

Baseline demographic, clinical, laboratory, immune, specimen, and in-hospital outcome variables are shown according to whether at least one structured pathogen was detected. Continuous variables are presented as median (interquartile range). Categorical variables are presented as number (percentage).

### 3.2 Pathogen spectrum

**Figure 2:**
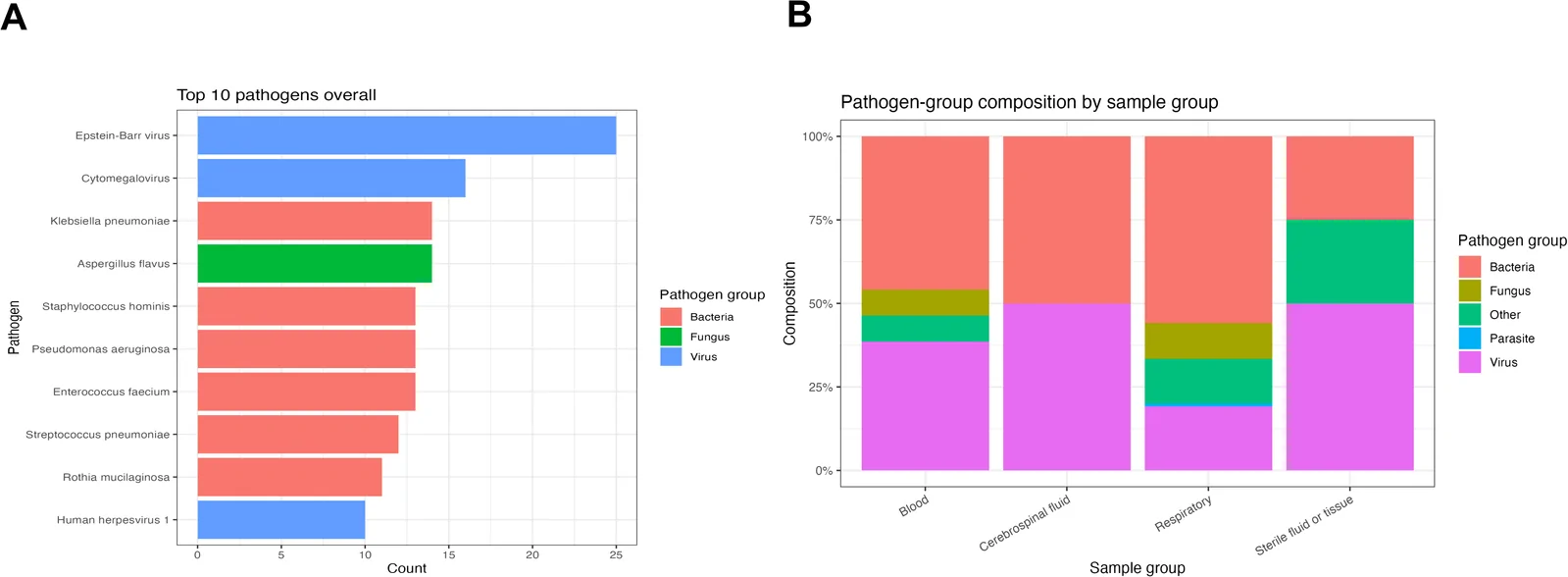
Overall pathogen spectrum and specimen-specific composition. Panel **(A)** shows the 10 most frequent pathogens after structured parsing and English standardization. Panel **(B)** shows the distribution of major pathogen classes across blood, respiratory, cerebrospinal fluid, sterile fluid or tissue, and other samples.

Bacteria were the most commonly detected pathogens, followed by viruses and fungi. The most frequently identified organisms were Epstein–Barr virus, cytomegalovirus, Aspergillus spp., Klebsiella pneumoniae, Enterococcus faecium, and Pseudomonas aeruginosa. Polymicrobial detections were common. The overall pathogen spectrum and the distribution of major pathogen groups by specimen type are shown in **Figure 2**.

### 3.3 Actionability-based reassessment

At the patient level, 78 of 134 patients (58.2%) had at least one high-confidence actionable finding. Twenty-one patients (15.7%) were classified as intermediate confidence, and 18 (13.4%) as low confidence. Seventeen patients (12.7%) had negative mNGS report.

**Figure 3:**
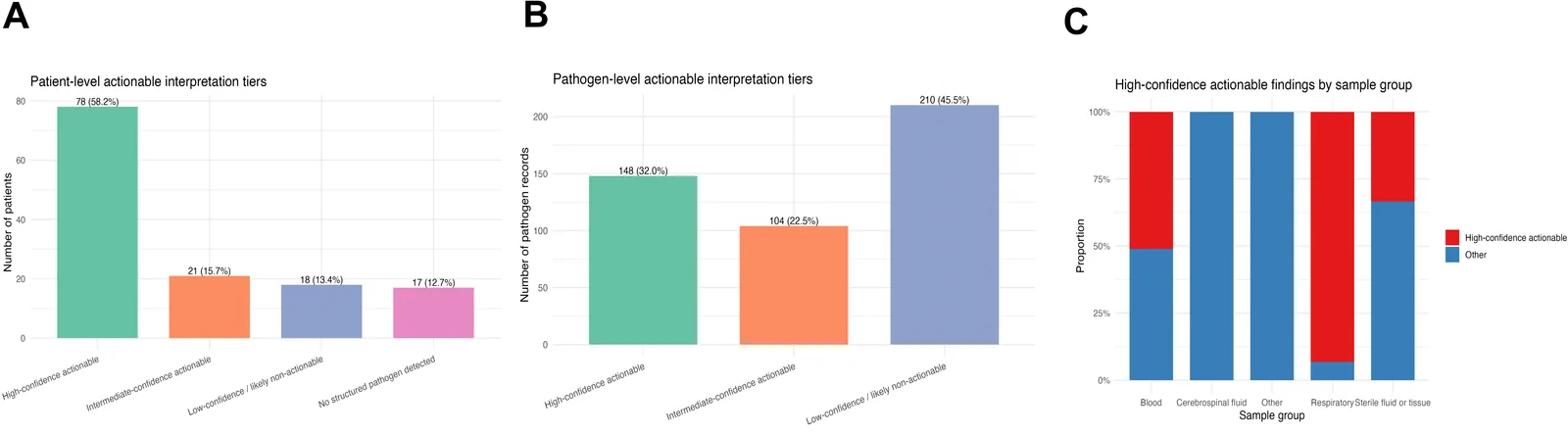
Reassessment of mNGS findings by clinical actionability. Panel **(A)** shows patient-level interpretation tiers. Panel **(B)** shows pathogen-level interpretation tiers. Panel **(C)** shows the proportion of patients with high-confidence actionable findings across specimen groups.

At the pathogen level, 462 organism records were identified. Of these, 148 (32.0%) were classified as high-confidence actionable, 104 (22.5%) as intermediate confidence, and 210 (45.5%) as low confidence or likely non-actionable. High-confidence actionable findings were more common in respiratory specimens than in blood samples. The distribution of interpretation tiers at both the patient and pathogen levels, as well as differences across specimen types, is shown in **Figure 3**. Detailed data are provided in **Table 3**.

**Table 3:** Action-ability based reassessment of mNGS findings.

3A. Patient-level interpretation tiers.				
Tier	Definition			Patients, n (%)
Tier 1	High-confidence actionable pathogen(s); directly guided therapy			78 (58.2%)
Tier 2	Intermediate-confidence actionable findings			21 (15.7%)
Tier 3	Low-confidence or uncertain clinical significance			18 (13.4%)
Tier 4	negative mNGS report			17 (12.7%)
Total				134

3B. Pathogen-level interpretation tiers.				
Tier	Definition			Number of pathogens, n (%)
Tier 1	Definite pathogen consistent with clinical presentation			148(32.0%)
Tier 2	Probable pathogen with partial supporting evidence			104(22.5%)
Tier 3	Unlikely pathogen (contaminant/colonizer)			210(45.5%)
Total				462

3C. Distribution of high-confidence actionable findings by specimen type.				
Specimen type	Total samples (n)	High-confidence actionable findings (n, %)		
Blood	98	50(51%)		
BALF	32	27(84.4%)		
Tissue	3	1(33.3%)		
Others	1	0		

3D. In-hospital outcomes by patient-level tiers.				
Patient actionable tier		Total samples (n)	Adverse outcome n (%)	n (%)
High-confidence actionable		78	22	28.2%
Intermediate-confidence actionable		21	5	23.8%
Low-confidence/likely non-actionable		18	9	50.0%
negative mNGS report		17	4	23.5%

This workbook includes patient-level and pathogen-level interpretation tiers, the distribution of high-confidence actionable findings by specimen group, and comparisons of in-hospital outcome and length of stay across patient-level tiers. Adverse outcome defined as discharge against medical advice. .

### 3.4 Clinical correlates of actionable findings

In-hospital outcomes did not differ significantly across patient-level actionability tiers (Fisher’s exact test, p = 0.262). Length of stay differed overall across groups (Kruskal–Wallis, p = 0.048), but pairwise comparisons did not show consistent significant differences.

**Figure 4:**
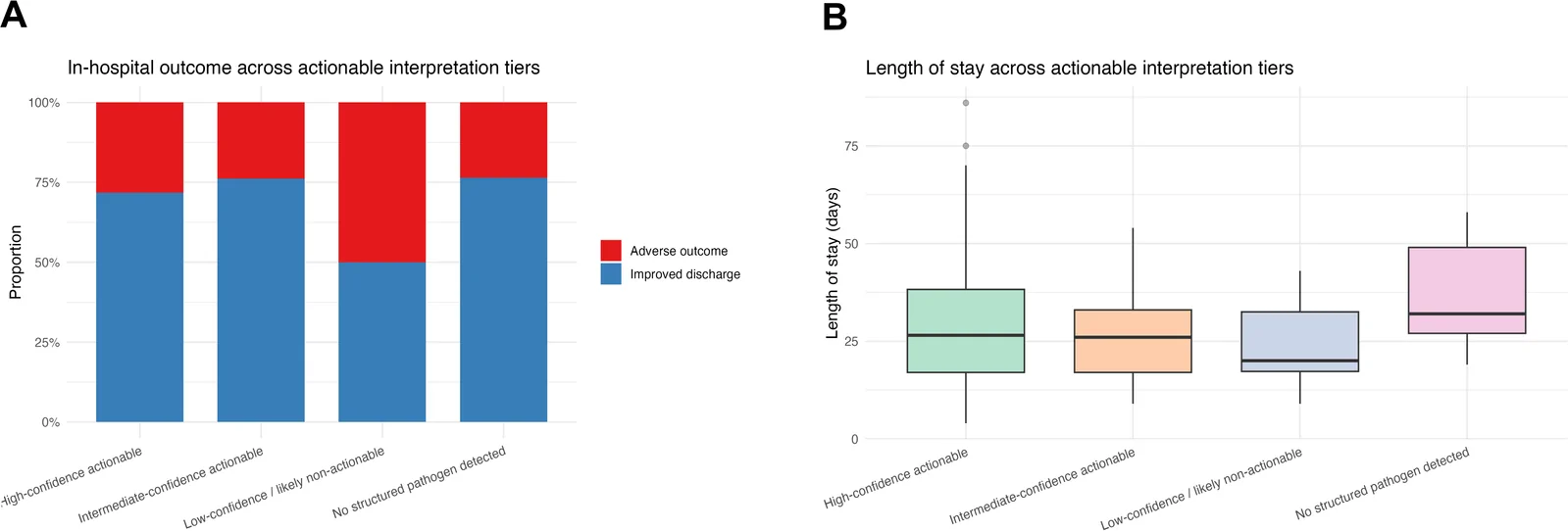
Short-term clinical relevance of actionable interpretation tiers. Panel **(A)** shows in-hospital outcome across patient-level actionable tiers. Panel **(B)** shows length of stay across actionable tiers. Overall comparisons were based on Fisher exact test and Kruskal-Wallis test.

In multivariable analysis, high-confidence actionable findings were not independently associated with adverse in-hospital outcomes (OR 0.67, 95% CI 0.27–1.59; p = 0.365). In contrast, older age remained an independent predictor of adverse outcome (OR 1.04 per year, 95% CI 1.02–1.07; p = 0.001). Outcome comparisons are shown in **Figure 4**.

### 3.5 Host correlates of fungal and viral detection

Fungal detection showed a distinct clinical pattern. Patients with fungal findings had higher neutrophil counts and were more frequently identified from non-blood specimens. Some differences in immune-cell parameters were also observed in immune-cell profiles, including the CD4/CD8 ratio. Overall, viral detection appeared to be associated with host immune characteristics and specimen source.

**Figure 5:**
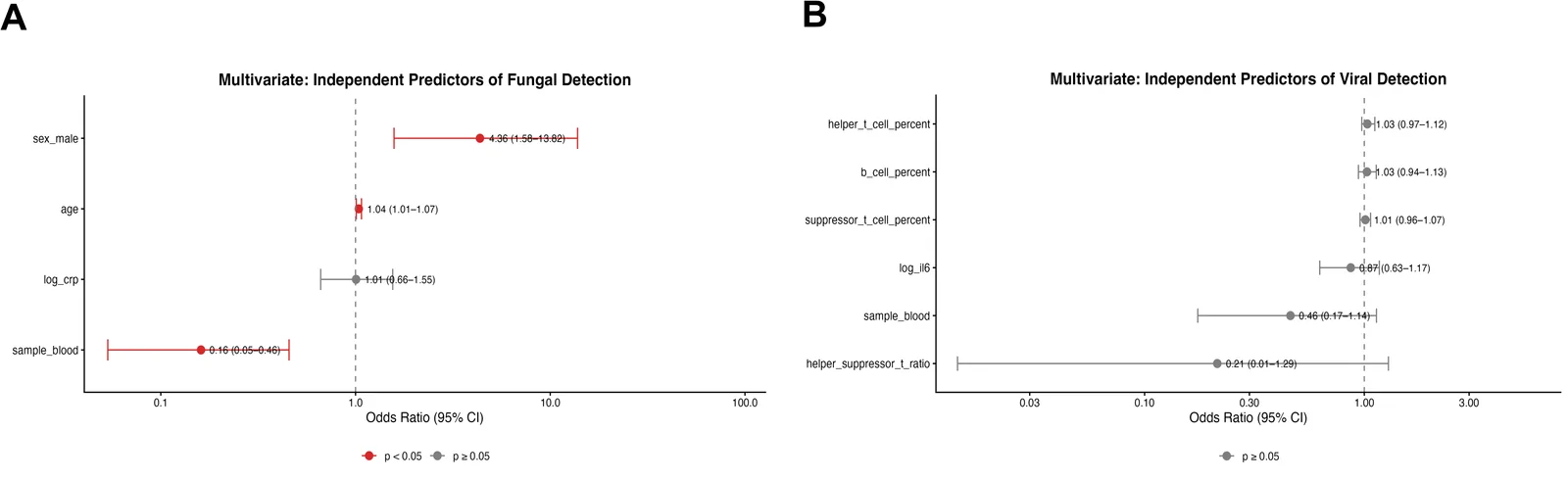
Host correlates of fungal and viral detection. Panel **(A)** shows the multivariable model for fungal detection. Panel **(B)** shows the multivariable model for viral detection. Odds ratios are shown with 95% confidence intervals.

Viral detection showed a different pattern. It was more closely related to host immune phenotype, especially imbalance in T-cell subsets. Comparative baseline characteristics are shown in **Tables 4**, **5**. Multivariable associations are shown in **Figure 5**.

**Table 4:** Baseline characteristics according to fungal detection, Comparison of baseline variables between patients with and without fungal detection on structured mNGS analysis.

Variable	Level	No	Yes	Overall	P-value
age		40.50 [30.00, 54.25]	51.50 [38.00, 62.75]	42.00 [33.00, 55.75]	0.02
length-of-stay-days		26.50 [18.00, 35.00]	28.50 [16.25, 42.75]	27.00 [17.25, 35.75]	0.78
WBC/10^9^		0.97 [0.25, 4.21]	4.36 [0.49, 7.94]	1.15 [0.30, 5.58]	0.04
NEUT/10^9^		0.52 [0.08, 2.27]	1.19 [0.07, 4.89]	0.61 [0.07, 2.95]	0.26
LYM/10^9^		0.33 [0.10, 0.77]	0.51 [0.16, 1.22]	0.35 [0.12, 0.85]	0.14
PCT		0.24 [0.15, 0.61]	0.21 [0.12, 0.59]	0.23 [0.14, 0.60]	0.38
CRP		69.14 [44.54, 120.55]	63.98 [17.30, 127.97]	69.04 [40.50, 123.52]	0.44
ESR		44.00 [36.00, 56.00]	44.00 [36.00, 55.00]	44.00 [36.00, 56.00]	0.67
IL6		89.20 [37.00, 145.00]	86.35 [33.32, 127.00]	89.20 [37.00, 145.00]	0.39
SF		1170.00 [980.00, 1372.50]	1215.00 [1000.00, 1417.50]	1170.00 [980.00, 1417.50]	0.82
Lymp%		74.40 [69.12, 77.60]	70.80 [67.80, 74.66]	72.20 [68.43, 77.10]	0.14
T-Cell		30.00 [15.70, 36.78]	30.00 [15.70, 35.22]	30.00 [15.70, 36.30]	0.85
T-cell%		42.00 [36.00, 59.14]	42.00 [34.47, 59.12]	42.00 [35.55, 59.25]	0.85
NK-cell		7.00 [5.64, 10.90]	9.79 [6.12, 13.62]	9.00 [5.71, 11.00]	0.18
B-cell%		5.80 [1.50, 7.00]	5.40 [1.50, 11.52]	5.60 [1.50, 7.40]	0.59
Helper-suppressor-T ratio		0.71 [0.27, 1.12]	0.71 [0.26, 1.03]	0.71 [0.26, 1.11]	0.69
CEA		1.88 [1.69, 2.12]	2.03 [1.76, 2.29]	1.88 [1.70, 2.12]	0.11
Sex	Female	48 (46.2%)	6 (20.0%)	54 (40.3%)	0.01
	Male	56 (53.8%)	24 (80.0%)	80 (59.7%)	NA
Disease Stage	Bone-Marrow-Suppression	23 (22.1%)	5 (16.7%)	28 (20.9%)	0.53
	Consolidation	2 (1.9%)	1 (3.3%)	3 (2.2%)	NA
	Induction-Remission	26 (25.0%)	7 (23.3%)	33 (24.6%)	NA
	Initial-Diagnosis	8 (7.7%)	3 (10.0%)	11 (8.2%)	NA
	Non-Remission	3 (2.9%)	0 (0.0%)	3 (2.2%)	NA
	Post-Transplant	23 (22.1%)	4 (13.3%)	27 (20.1%)	NA
	Refractory	6 (5.8%)	2 (6.7%)	8 (6.0%)	NA
	Relapse	6 (5.8%)	2 (6.7%)	8 (6.0%)	NA
	Remission	1 (1.0%)	2 (6.7%)	3 (2.2%)	NA
	Unknown	6 (5.8%)	4 (13.3%)	10 (7.5%)	NA
Initial diagnosis Flag	No	70 (67.3%)	20 (66.7%)	90 (67.2%)	1
	Yes	34 (32.7%)	10 (33.3%)	44 (32.8%)	NA
In hospital outcome	automatic-discharge	30 (28.8%)	8 (26.7%)	38 (28.4%)	0.89
	Death	2 (1.9%)	0 (0.0%)	2 (1.5%)	NA
	Improved-discharge	72 (69.2%)	22 (73.3%)	94 (70.1%)	NA
Sample group	blood	85 (81.7%)	13 (43.3%)	98 (73.1%)	0
	CSF	3 (2.9%)	0 (0.0%)	3 (2.2%)	NA
	other	1 (1.0%)	0 (0.0%)	1 (0.7%)	NA
	respiratory	12 (11.5%)	17 (56.7%)	29 (21.6%)	NA
	sterile-fluid-or-tissue	3 (2.9%)	0 (0.0%)	3 (2.2%)	NA

**Table 5:** Baseline characteristics according to viral detection, Comparison of baseline variables between patients with and without viral detection on structured mNGS analysis.

Variable	Level	No	Yes	Overall	P-value
Age		42.50 [34.00, 54.75]	41.50 [33.00, 58.25]	42.00 [33.00, 55.75]	0.6257
Length-of-stay-days		28.50 [20.00, 39.25]	26.00 [16.75, 34.00]	27.00 [17.25, 35.75]	0.1185
WBC/10^9^		0.76 [0.20, 4.04]	1.93 [0.40, 6.07]	1.15 [0.30, 5.58]	0.0692
NEUT/10^9^		0.23 [0.06, 1.30]	1.23 [0.08, 4.01]	0.61 [0.07, 2.95]	0.0292
Lymp/10^9^		0.27 [0.09, 0.73]	0.38 [0.16, 0.86]	0.35 [0.12, 0.85]	0.3106
PCT(ng/ml)		0.29 [0.16, 0.73]	0.20 [0.13, 0.40]	0.23 [0.14, 0.60]	0.1297
CRP		66.36 [46.08, 123.33]	70.82 [38.38, 123.14]	69.04 [40.50, 123.52]	0.9656
ESR		46.00 [37.00, 56.00]	44.00 [36.00, 52.00]	44.00 [36.00, 56.00]	0.3446
IL6		109.00 [51.00, 163.00]	73.00 [34.18, 133.10]	89.20 [37.00, 145.00]	0.0864
SF		1215.00 [1000.00, 1440.00]	1170.00 [980.00, 1350.00]	1170.00 [980.00, 1417.50]	0.6033
T-lymphocyte(%)		74.97 [69.20, 77.73]	71.40 [68.40, 76.40]	72.20 [68.43, 77.10]	0.169
Helper-t-cell-percent		30.70 [27.93, 43.81]	29.30 [15.13, 34.70]	30.00 [15.70, 36.30]	0.0921
Suppressor-t-cell-percent		40.60 [35.47, 45.81]	42.70 [35.85, 59.30]	42.00 [35.55, 59.25]	0.0602
NK-cell-percent		7.00 [5.56, 11.00]	9.25 [5.92, 11.00]	9.00 [5.71, 11.00]	0.4885
B-cell-percent		6.40 [2.54, 8.04]	5.20 [1.25, 6.85]	5.60 [1.50, 7.40]	0.0542
Helper-suppressor-t-ratio		0.72 [0.70, 1.21]	0.71 [0.25, 1.03]	0.71 [0.26, 1.11]	0.0186
CEA		1.88 [1.68, 2.12]	1.94 [1.70, 2.15]	1.88 [1.70, 2.12]	0.5232
Sex	Female	22 (40.7%)	32 (40.0%)	54 (40.3%)	
	Male	32 (59.3%)	48 (60.0%)	80 (59.7%)	
Disease-Stage	Bone-Marrow-Suppression	14 (25.9%)	14 (17.5%)	28 (20.9%)	0.768
	Consolidation	1 (1.9%)	2 (2.5%)	3 (2.2%)	NA
	Induction-Remission	15 (27.8%)	18 (22.5%)	33 (24.6%)	NA
	Initial-Diagnosis	4 (7.4%)	7 (8.8%)	11 (8.2%)	NA
	Non-Remission	1 (1.9%)	2 (2.5%)	3 (2.2%)	NA
	Post-Transplant	8 (14.8%)	19 (23.8%)	27 (20.1%)	NA
	Refractory	5 (9.3%)	3 (3.8%)	8 (6.0%)	NA
	Relapse	2 (3.7%)	6 (7.5%)	8 (6.0%)	NA
	Remission	1 (1.9%)	2 (2.5%)	3 (2.2%)	NA
	Unknown	3 (5.6%)	7 (8.8%)	10 (7.5%)	NA
Initial-diagnosis-flag	No	35 (64.8%)	55 (68.8%)	90 (67.2%)	0.7087
	Yes	19 (35.2%)	25 (31.2%)	44 (32.8%)	NA
In-hospital-outcome	automatic-discharge	19 (35.2%)	19 (23.8%)	38 (28.4%)	0.2316
	Death	0 (0.0%)	2 (2.5%)	2 (1.5%)	NA
	Improved-discharge	35 (64.8%)	59 (73.8%)	94 (70.1%)	NA
Sample-group	Blood	44 (81.5%)	54 (67.5%)	98 (73.1%)	0.038
	CSF	1 (1.9%)	2 (2.5%)	3 (2.2%)	NA
	other	1 (1.9%)	0 (0.0%)	1 (0.7%)	NA
	respiratory	6 (11.1%)	23 (28.8%)	29 (21.6%)	NA
	sterile-fluid-or-tissue	2 (3.7%)	1 (1.2%)	3 (2.2%)	NA

### 3.6 Exploratory analyses of pathogen burden

**Figure 6:**
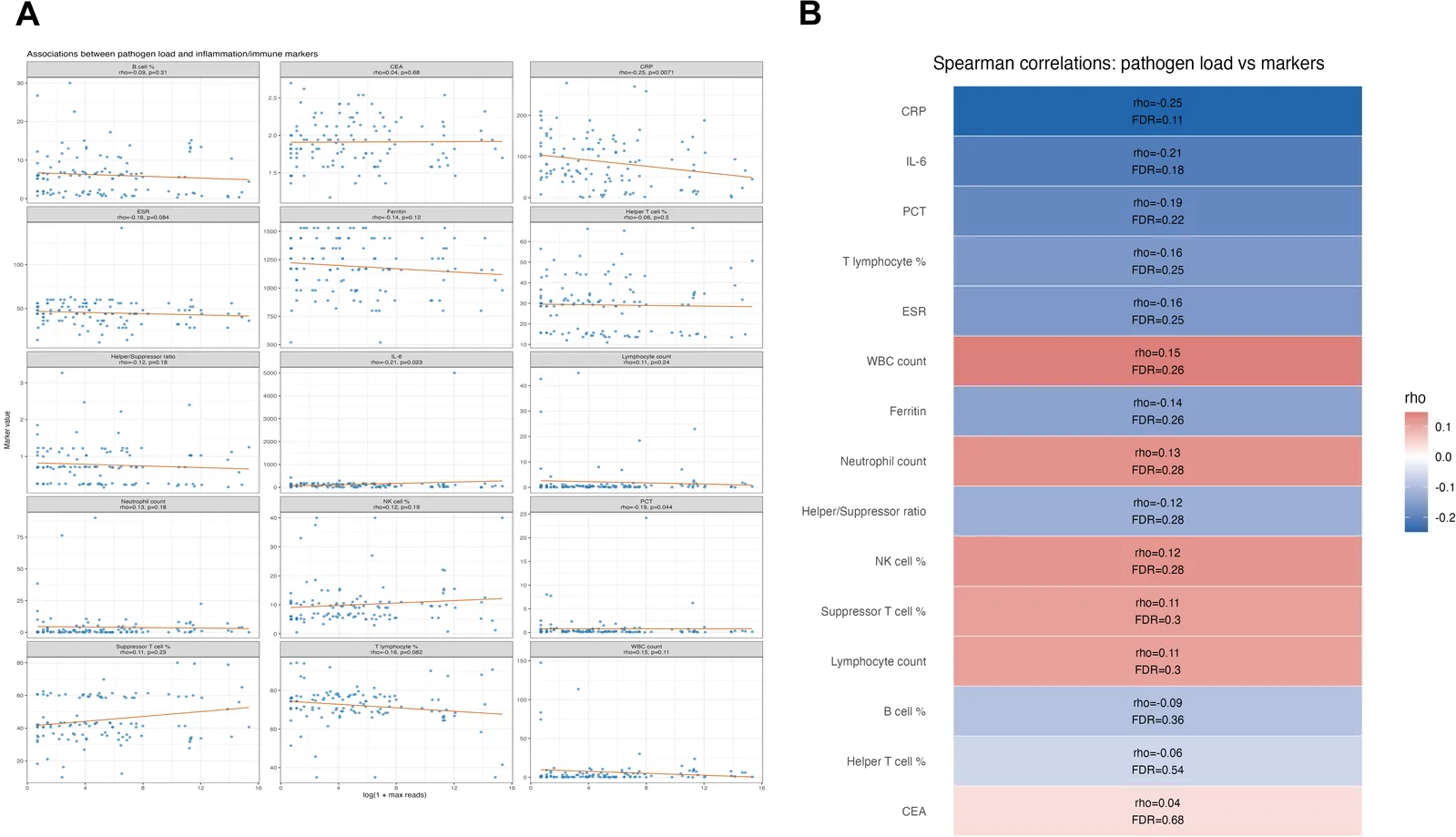
Exploratory associations between pathogen burden and host markers. Panel **(A)** shows faceted plots of log-transformed maximum read count against inflammatory and immune markers. Panel **(B)** shows the correlation heatmap for the same marker set. These analyses were exploratory.

Read-based analyses were exploratory. Pathogen read counts showed weak inverse correlations with inflammatory markers, including CRP, IL-6, and PCT, although none remained significant after FDR correction. In multivariable linear regression, IL-6 remained independently associated with pathogen read counts (β = 30.34, 95% CI 4.78–55.90; P = 0.022), whereas other laboratory markers showed no significant associations. The detailed results are presented in **Table 6**. Pathogen read burden was not independently associated with adverse in-hospital outcomes. Its association with length of stay was inconsistent across models. These exploratory findings are shown in **Figure 6**, with statistical details provided in **Table 7**.

**Table 6:** Factors associated with mNGS reads: correlation and linear regression analyses.

6A				
Variable	n	Spearman ρ	P value	FDR-adjusted P
CRP (mg/L)	116	-0.249	0.007	0.107
IL-6 (pg/mL)	116	-0.21	0.023	0.176
PCT (ng/mL)	116	-0.187	0.044	0.221
T lymphocytes (%)	116	-0.162	0.082	0.252
ESR	116	-0.161	0.084	0.252
WBC count (×10^9^/L)	116	0.147	0.114	0.262
Ferritin	116	-0.144	0.122	0.262
Neutrophil count (×10^9^/L)	116	0.126	0.179	0.282

6B				
Variable			β (95% CI)	P value
IL-6 (pg/mL)			30.34 (4.78 ~ 55.90)	0.022
Suppressor T cells (%)			0.67 (-0.02 ~ 1.36)	0.06
T lymphocytes (%)			-0.43 (-0.98 ~ 0.12)	0.132
WBC count (×10^9^/L)			-0.80 (-1.92 ~ 0.32)	0.167
CRP (mg/L)			-1.04 (-4.33 ~ 2.24)	0.535
PCT (ng/mL)			0.06 (-0.08 ~ 0.19)	0.438
Neutrophil count (×10^9^/L)			-0.08 (-0.71 ~ 0.55)	0.815
Lymphocyte count (×10^9^/L)			-0.17 (-0.56 ~ 0.22)	0.394
ESR			-0.15 (-0.99 ~ 0.70)	0.732
Ferritin			0.24 (-12.88 ~ 13.36)	0.971
Helper T cells (%)			0.21 (-0.47 ~ 0.89)	0.549
NK cells (%)			-0.02 (-0.42 ~ 0.38)	0.915
B cells (%)			0.01 (-0.30 ~ 0.31)	0.968
CD4/CD8 ratio			-0.004 (-0.03 ~ 0.02)	0.777
CEA			-0.001 (-0.014 ~ 0.011)	0.822

Spearman correlation (with Benjamini–Hochberg FDR correction) and linear regression were used to assess associations between mNGS reads and laboratory markers, with results reported as β coefficients and 95% CIs.

**Table 7:** Multivariable logistic regression analysis of factors associated with actionable mNGS findings.

Variable	OR (95% CI)	P value
Sample type (blood)	0.46 (0.18–1.14)	0.102
Log (IL-6)	0.87 (0.63–1.17)	0.366
Helper T cell (%)	1.03 (0.97–1.12)	0.347
Suppressor T cell (%)	1.01 (0.96–1.07)	0.682
B cell (%)	1.03 (0.94–1.13)	0.549
Helper/Suppressor T ratio	0.21 (0.01–1.29)	0.169

Odds ratios (ORs) with 95% confidence intervals (CIs) were estimated using multivariable logistic regression to identify factors independently associated with actionable mNGS findings.

## 4 Discussion

This study highlights the need to interpret mNGS results in clinical context in hematologic practice. Three main findings emerged. First, the overall positivity rate was high, but only part of the detected signal remained clinically actionable after structured review. Second, specimen type had a strong support on apparent clinical yield. Third, fungal and viral detections showed different host associations, while read burden alone had limited value for short-term prognostic assessment.

The high positivity rate was not surprising. Broad-range sequencing can detect a wide range of microbial signals in immunocompromised patients (Chiu and Miller, 2019; Gu et al., 2019; Han et al., 2019). Earlier studies in hematologic and other immunocompromised populations have also reported improved diagnostic yield with mNGS (Zinter et al., 2019; Shen et al., 2021; Wang et al., 2022; Zhang et al., 2022; Yuan et al., 2024). Our results are in line with those reports. At the same time, they reinforce a well-known limitation of mNGS: a positive result does not always carry the same clinical significance. After contextual review, a substantial proportion of organism-level findings fell into the low-confidence or likely non-actionable category. This agrees with previous studies showing that metagenomic results often require careful clinical assessment before they can inform management (Hogan et al., 2021; Lehman et al., 2024; Vinh Dong et al., 2024; Yin et al., 2025).

This issue is potentially actionable relevant to routine hematology practice. Clinicians often encounter mixed microbial findings, herpesvirus reactivation, and signals that may reflect colonization rather than invasive disease. For this reason, a simple positive-versus-negative interpretation is often inadequate. Our tiered model was designed to better connect sequencing output with bedside decision-making. More than half of the patients had at least one high-confidence actionable finding, suggesting that mNGS can provide clinically useful information in a substantial subset of cases.

Specimen context was one of the strongest factors affecting apparent clinical yield. High-confidence actionable findings were much more common in respiratory samples than in blood samples. This difference is clinically understandable. Respiratory specimens are often obtained in the setting of a more clearly defined syndrome, whereas blood-based testing may capture a broader and less specific pool of circulating microbial DNA. Because this study analyzed finalized clinical mNGS reports rather than raw sequencing data, oral commensal organisms may be underrepresented in respiratory specimens due to routine clinical reporting practices, rather than assay-level suppression or specimen-specific filtering. Similar concerns have been reported in studies of plasma metagenomics (Chiu and Miller, 2019; Gu et al., 2019; Han et al., 2019; Hogan et al., 2021; Lehman et al., 2024; Vinh Dong et al., 2024; Yin et al., 2025).

Fungal findings deserve special attention. In our cohort, fungal detection was associated with older age and was more common in non-blood specimens. In univariable analyses, fungal detection was also associated with higher white blood cell counts, although this result should be interpreted with caution. These findings are broadly consistent with clinical experience in hematologic patients and transplant recipients, in whom invasive fungal disease remains challenging to diagnose and manage (Alexander et al., 2021; Dadwal et al., 2021). Importantly, fungal detection was not significantly associated with length of stay in our data. This suggests that it may reflect underlying host status or diagnostic practice more than a direct effect on hospitalization duration.

Viral detection showed a different pattern. It was more closely related to host immune phenotype, especially imbalance in T-cell subsets. These associations were modest and should be regarded as exploratory. This interpretation is also compatible with clinical practice, where Epstein-Barr virus (EBV) or Human cytomegalovirus (CMV) detection may indicate immune dysregulation or viral reactivation rather than a typical acute inflammatory process.

Read burden was less informative than we initially expected. Its correlations with inflammatory markers were weak, and it was not independently associated with short-term outcome. These findings argue against treating raw read counts as a direct marker of disease severity. Read counts can be affected by specimen type, microbial biology, sequencing depth, background contamination, and timing of sampling.

This study has several limitations. First, it was a retrospective single-center study with a modest sample size. Some subgroup analyses were unstable because of sparse data, and in-hospital death was uncommon. The small number of adverse outcome events limited the power of multivariable analyses and increased the risk of model instability and overfitting. Second, the actionability framework was rule-based and was not formally validated against adjudicated clinical endpoints that incorporated conventional microbiology or treatment response. Furthermore, because conventional microbiological investigations were not performed uniformly across all patients, we were unable to directly compare mNGS with conventional diagnostic methods in terms of diagnostic concordance, time to microbiological diagnosis, or alternative diagnoses in patients with negative mNGS results. Third, although variables in multivariable models were selected on clinical grounds, residual confounding and possible multicollinearity cannot be excluded. Validation in independent cohorts is still needed.

Another limitation is the imbalance in specimen distribution, with blood samples accounting for the majority of mNGS tests. This pattern largely reflects real-world clinical practice, as blood is often the initial specimen selected in hematologic patients with suspected systemic infection or febrile episodes due to its accessibility, standardized collection procedures, and routine availability. Consequently, the observed pathogen spectrum and actionability profile may have been influenced by this sampling pattern and may not fully represent infections occurring at non-sterile sites, particularly the respiratory tract. This imbalance may also partly explain the lower proportion of high-confidence actionable findings identified in blood samples compared with respiratory specimens in our cohort. In addition, some clinically relevant variables, including detailed antimicrobial exposure and neutropenia status at the time of mNGS sampling, were not consistently documented in this retrospective dataset, which limited further stratified analyses and may have introduced residual confounding.

Future studies should include a more balanced range of specimen types to test whether these findings are generalizable. Prospective designs with standardized sampling strategies and predefined clinical adjudication criteria would also improve the interpretability of mNGS results in this population.

Despite these limitations, the study still has practical relevance. It shifts attention from mNGS positivity alone to clinical interpretation of the result and offers a pragmatic tiered framework that may help integrate mNGS findings into routine hematology care. Because of the limited number of adverse outcome events, multivariable analyses should be considered exploratory and interpreted with caution.

## 5 Conclusion

In hospitalized patients with hematologic diseases, first-time mNGS testing had a high detection rate, but only part of the signal remained clinically actionable after structured review. The clinical value of mNGS depended more on interpretation than on positivity alone. Specimen type affected clinical yield, with respiratory samples more often showing clearly relevant findings. Fungal and viral detections had different host associations, while read burden showed limited prognostic value. These results support interpreting mNGS findings in the context of specimen type, host status, and organism plausibility.

## Data Availability

The datasets presented in this study can be found in online repositories. The names of the repository/repositories and accession number(s) can be found in the article/supplementary material.
